# Associations Between Energy Balance-Related Behaviours and Childhood Obesity Among Vulnerable Populations in Greece: Implications for Public Health Policy and Intervention Development

**DOI:** 10.3390/nu17213486

**Published:** 2025-11-06

**Authors:** George Moschonis, Anela Halilagic, Matzourana Argyropoulou, Theodora Balafouti, Renos Roussos, Vaios Svolos, Pauline Dacaya, Odysseas Androutsos, Theodora Mouratidou, Yannis Manios

**Affiliations:** 1 Department of Food, Nutrition and Dietetics, School Allied Health, Human Services & Sport, La Trobe University, Plenty Rd, Kingsbury Dr, Bundoora, Melbourne, VIC 3086, Australia; a.halilagic@latrobe.edu.au (A.H.); paulinedacaya@gmail.com (P.D.); 2 Department of Nutrition and Dietetics, School of Health Sciences and Education, Harokopio University of Athens, 17676 Kallithea, Greece; margyropoulou@hua.gr; 3 Department of Nutrition and Dietetic Sciences, School of Health Sciences, Hellenic Mediterranean University, 72300 Sitia, Greece; tbalafouti@hmu.gr (T.B.); rroussos@med.uoa.gr (R.R.); tmouratidou@hmu.gr (T.M.); 4 School of Medicine, Dentistry and Nursing, College of Medical, Veterinary and Life Sciences, University of Glasgow, Glasgow G12 8QQ, UK; vaiosvol@uth.gr; 5 Lab of Clinical Nutrition and Dietetics, Department of Nutrition and Dietetics, School of Physical Education, Sports Science and Dietetics, University of Thessaly, 42100 Trikala, Greece; oandroutsos@uth.gr; 6 Institute of Agri-Food and Life Sciences, University Research & Innovation Center, Hellenic Mediterranean University, 71003 Crete, Greece

**Keywords:** children, overweight, obesity, behaviour, vulnerable, energy-balance

## Abstract

**Background/Objectives**: Childhood obesity remains a critical public health concern in Greece, particularly among socioeconomically vulnerable groups. This study conducted a secondary analysis of five large-scale epidemiological datasets to examine the association between energy balance-related behaviours (EBRBs) and obesity in children in need. **Methods**: Data were compiled from five nationally or regionally representative studies (Genesis, ToyBox, Healthy Growth, ENERGY, and Feel4Diabetes) involving children aged 1–12 years. Stratified and subgroup analyses were performed to examine associations between weight status and EBRBs, including dietary habits, physical activity, and sedentary behaviour. Determinants of EBRBs were also analysed using the socio-ecological model framework. **Results**: Children in need demonstrated a higher prevalence of overweight and obesity compared to the general child population. Key risk factors for EBRBs included frequent consumption of sugar-sweetened beverages, sweet snacks, and high screen time. Protective behaviours associated with lower obesity risk included regular breakfast consumption, adequate sleep duration, and physical activity. Determinants of high-risk EBRBs were primarily interpersonal and, to a lesser extent, individual and community-level factors. **Conclusions**: These findings highlight the disproportionate burden of childhood obesity among vulnerable populations and identify modifiable behaviours and determinants that can inform targeted interventions. These results provide a robust evidence base to guide national public health policies, including the development of school- and community-based obesity prevention programmes aligned with the goals of Greece’s National Action Against Childhood Obesity. Prioritising children in need in such initiatives is essential to reduce health inequities and improve long-term health outcomes.

## 1. Introduction

Obesity is a multifactorial disease that was once considered a concern predominantly affecting high-income countries. However, the obesity epidemic has expanded globally with low- and middle-income countries now experiencing a significant rise in populations living with overweight or obesity [1]. The World Health Organization (WHO) highlighted a concerning increase in the global prevalence of overweight and obesity among children and adolescents over the past three decades, rising from 2% to 8% [2]. Recent reports indicate that 35 million children under the age of 5 and 390 million children and adolescents aged 5 to 19 years are currently overweight [2]. Furthermore, the World Obesity Federation projects that between 2020 and 2035, the percentage of boys and girls living with obesity will increase by 61% and 75%, respectively [3].

Being overweight during childhood and adolescence is associated with immediate physical health consequences and an increased risk of early-onset chronic conditions, such as type 2 diabetes and cardiovascular disease [4]. Children living with obesity are also more likely to become adults living with obesity, perpetuating long-term health impacts [4]. Overweight, obesity, and associated non-communicable diseases are largely preventable and manageable through effective public health strategies [4]. The psychosocial impact of obesity in childhood is also well-documented, with effects on schooling and quality of life intensified by bullying and stigmatisation [4]. In addition, the WHO has underscored the significant economic burden of obesity, with global costs projected to reach $3 trillion (US) by 2030 if current trends persist [2].

In 2024, the WHO European Childhood Obesity Surveillance Initiative (COSI) released data from 37 European countries, covering 470,000 children. According to this report, 27% of boys and 23% of girls were classified as overweight, while 13% of boys and 9% of girls were living with obesity [5]. Compared to previous COSI reports, a vast majority of countries were unable to reduce their prevalence of overweight and obesity. These findings indicate that excess body weight remains a pressing public health concern across the region [5].

Some countries, including Bulgaria, Malta, Slovenia, and Sweden, have reported an increase in the prevalence of overweight among children, while others, including Greece, Italy, and Spain, have shown a decline [5]. However, despite the reductions, the prevalence of overweight and obesity remains highest in southern European countries [6]. Greece continues to rank among the countries with the highest rates of childhood obesity in Europe, with one in three children classified as overweight or obese across multiple age groups [7]. The issue has been further compounded by the economic downturn following the 2008 recession, which led to increased poverty levels and worsened health outcomes, particularly among vulnerable population groups in Greece [8].

Obesity is a complex condition shaped by a combination of obesogenic environments, psychosocial factors, and genetic predispositions [9]. One key aspect of the obesogenic environment is the lack of structural supports that enable access to healthy, affordable food, particularly for disadvantaged populations [1]. As such, the responsibility for addressing obesity lies with society rather than the individual. Creating supportive environments that promote healthy eating and regular physical activity as accessible and affordable lifestyle options is essential for long-term prevention [2]. Several behaviours related to energy-balance, commonly referred to as energy balance-related behaviours (EBRB), play a critical role in obesity prevention. These include dietary habits, physical activity, and sedentary behaviours [10].

Obesity rates are rising rapidly in low- and middle-income countries, particularly among populations with lower socioeconomic status (SES) [1]. Social determinants of health, including the conditions in which people are born, grow, live, and work, can either support or undermine the health of individuals and communities [11]. As a result, social inequalities often contribute to unjust disparities in health outcomes across population groups [11]. Indicators of socioeconomic disadvantage, such as lower income and educational attainment, are associated with increased energy intake and reduced energy expenditure, thereby increasing the risk of obesity among those with lower SES [12]. Higher levels of education attainment have consistently been linked to a lower likelihood of being overweight or obese [11]. Family SES is a particularly influential role in childhood obesity, with lower SES associated with a greater risk [13]. Key indicators of SES in children include parental education, household income, and residential setting (i.e., urban vs. rural) [14].

Children are widely considered the most vulnerable age group, with those facing increased disadvantages often referred to as children in need [8]. The European Union defines this group to include homeless children or those experiencing severe housing deprivation, children with disabilities or mental health issues, children from migrant backgrounds or minority ethnic groups, those in alternative (especially institutional) care, and children living in precarious family situations [8]. The association between SES and overweight and obesity is well-established. Evidence indicates that children in Greece are among the most at risk of poverty and social exclusion in Europe, factors that significantly contribute to their vulnerability to overweight and obesity [8].

The primary objective of the current study was to conduct a secondary statistical analysis of available data on children in need in Greece, defined as those with low SES, based on indicators such as low parental education, unemployment, ethnic minority background, and residence in rural, urban, or metropolitan areas. The aim was to identify EBRBs associated with overweight and obesity within this population.

The secondary objective was to further explore the data to identify the most significant determinants of these EBRBs among children in need. This included examining personal characteristics (e.g., social norms, attitudes, perceptions, parental or carer practices, and gender), as well as broader social and physical environmental factors.

## 2. Materials and Methods

### 2.1. Study Design

This secondary data analysis was based on data from five studies involving children, adolescents, and their families living in different regions of Greece. These included two sub-national cross-sectional studies (i.e., Genesis and Healthy Growth study) and three EU-funded intervention studies (i.e., ToyBox, Energy, and Feel4Diabetes study), from which baseline cross-sectional data collected in Greece was utilised. All studies received ethical approval from the Bioethics Committee of Harokopio University and were conducted in full compliance with the Declaration of Helsinki and the conventions of the Council of Europe on human rights and biomedicine. A meta-analysis was not performed. The secondary analyses were conducted on datasets independently and not pooled.

Table 1 summarises the main characteristics of the five studies, including the age range of the children involved, the population, the total sample size, years, duration, and the geographic locations in Greece where data was collected.

### 2.2. Sampling

The Genesis study was conducted from April 2003 to July 2004 with a population of toddlers and preschool children in Greece, aged between 1 and 5 years [15]. A representative number of randomly selected public and private nurseries, as well as day-care centres, within municipalities in four regions (namely Attica, Etoloakarnania, Thessaloniki, Halkidiki) were invited to participate in the study. Furthermore, an extended letter explaining the aims of the current study and a consent form were provided to the parents whose child was in these nurseries. From the total number of positive responses, complete data became available for 2374 children, with participation rates varying from 54% to 95%, with the highest rates in rural areas and the lowest in urban areas. More information on the sampling procedures is presented elsewhere [15].

The ToyBox study was a multicomponent, kindergarten-based, family-involved intervention with a cluster-randomised design, which focused on modifying EBRBs to prevent obesity [16]. More information on the sampling procedures is presented elsewhere [16]. A standardised, multistage sampling approach was applied to identify preschool children aged 3.5–5.5 years old and their families, who were recruited from randomly selected kindergartens within randomly selected municipalities of Attica. Five municipalities were randomly selected from each SES tertile, and a list of all kindergartens located within each of these randomly chosen municipalities was created. An extended letter explaining the aims of the current study and a consent form were provided to each parent with a child in these kindergarten classes. Parents who agreed to participate in the study had to sign a consent form and provide their contact details. From the total number of positive responses, complete data became available for 1229 children from Greece.

The Feel4Diabetes study was a large school- and community-based study aiming to promote a healthy lifestyle in families at risk for type 2 diabetes in Europe [17]. The actual implementation of the Feel4Diabetes study had a total duration of two years (2016–2018). The municipalities in Attica were then grouped into tertiles according to socioeconomic level, according to census data retrieved from the Hellenic Statistical Authority. A list of all primary schools within the randomly selected vulnerable areas was created, and primary schools were randomly selected and recruited from each area until the recruitment goal was met. Children attending the first three grades within these primary schools were recruited after providing a detailed letter to their parents explaining the study objectives and all participation requirements. Signed parental consent forms were collected for 2282 families.

The Healthy Growth study was a large-scale cross-sectional epidemiological study initiated in May 2007 and completed in June 2009 [18]. In addition to ethics approval previously described, approval to conduct the study was also granted by the Greek Ministry of National Education. The population under study mostly comprised school-aged children between 10 and 12 years, attending the 5th and 6th grades of primary schools located in municipalities within the prefectures of Attica, Etoloakarnania, Thessaloniki, and Heraklion. The sampling of schools was random, multistage, and stratified by parental education level and total population of students attending schools within these municipalities. Signed parental consent forms were collected for 2655 out of 4145 children (response rate 64.1%). From the total number of positive responses, complete data became available for 2294 children.

The Energy study was conducted among 10- to 12-year-old children and their parents, with data collection completed between March and July 2010 [19]. As detailed elsewhere, the sampling of schools was random, multistaged, and stratified by degree of urbanisation in the under-study regions in Athens [19]. Following the school’s approval for participation in the study, parents received a letter explaining the study’s purpose and were asked to provide written consent for participation. A total of 37 schools participated, with data collected for 1077 children and their parents (response rate 60%).

### 2.3. Outcome Variables

#### 2.3.1. Socio-Ecological Framework

The identification of determinants of behaviours related to energy balance and weight status in children and adolescents in the present study was guided by the socio-ecological framework [20,21]. This framework recognises that health behaviours are influenced by multiple, interacting factors operating at individual, interpersonal, organisational, community, and policy levels. At the individual level, factors such as age, sex, knowledge, and self-efficacy affect eating and activity behaviours. The interpersonal level involves influences from parents, peers, and caregivers who shape dietary habits, physical activity, and sedentary patterns through role modelling and support. The organisational level includes factors within schools and other institutional settings, such as food provision, opportunities for physical activity, and relevant policies. The community level encompasses neighbourhood characteristics, including access to recreational spaces and food environments, while the policy level refers to broader regulatory, economic, and social policies that shape the availability and promotion of healthy choices. By acknowledging these interrelated levels of influence, the socio-ecological framework provides a comprehensive basis for identifying multilevel determinants of behaviours contributing to energy balance and weight status in the paediatric population.

#### 2.3.2. Anthropometric and Weight Status Variables

Variables related to children’s weight status served as dependent variables in the examination of the primary objective of this secondary data analysis. Children’s body weight (kg), height (cm), body mass index (BMI, kg/m^2^), BMI-for-age z-score, and waist circumference (cm) were assessed across studies. BMI z-scores were calculated according to the WHO Growth Standards [22], and BMI categories were defined using International Obesity Task Force (IOTF) age- and sex-specific cut-off points to classify children as underweight, normal weight, overweight, or obese [23,24]. Waist circumference categories were defined using age- and sex-specific percentile thresholds, with central obesity defined as a waist circumference greater than or equal to the 90th percentile. Table S1 in the Supplementary Materials lists all continuous and categorical variables related to children’s weight status by study.

#### 2.3.3. Energy Balance-Related Behaviours (EBRBs)

All available continuous and categorical variables related to children’s EBRBs by study served as independent variables in the examination of the primary objective and as dependent variables in the examination of the secondary objective of this secondary data analysis. Table S2 of this report can be found in the Supplementary Materials and lists all continuous and categorical variables related to children’s EBRBs by study.

Across studies, children’s EBRBs were assessed through validated questionnaires or recalls capturing dietary intake, physical activity (PA), screen time, and sleep. Dietary intake (continuous variables) included total daily energy intake (kcal/day) and consumption frequencies of food groups such as fruits, vegetables, dairy, whole-grains, fish, and sugar-sweetened beverages (servings/day or times/week). Corresponding categorical variables reflected habitual food intake frequencies (e.g., never, 1–2 times/week, ≥5 times/week). Physical activity was operationalised as light-to-vigorous or moderate-to-vigorous intensity PA (min/day or steps/day), derived from accelerometer or questionnaire data, depending on the study. In categorical analyses, adequate activity was defined as achieving greater than or equal to 60 min of moderate-to-vigorous PA per day, consistent with WHO recommendations [25]. Sedentary behaviour included time spent watching television, using computers or other screens (hours/day on weekdays and weekends). In categorical form, excessive sedentary time was defined as >2 h/day of screen use, aligned with international PA and sedentary guidelines for children [25]. Sleep duration was assessed as average hours/day (continuous) and categorised according to age-specific recommendations (e.g., adequate sleep defined as greater than or equal to 9 h per night). Both continuous and categorical variables are listed in Supplementary Materials Tables S1 and S2.

Table S3 in the Supplementary Materials presents those continuous and categorical variables that were examined to assess their potential role as determinants of children’s EBRBs by study. These variables served as independent variables in the examination of the secondary objective of this secondary data analysis.

Table S4 in the Supplementary Materials presents those socioeconomic status variables that were used for the identification of vulnerable populations of children ‘in need’ in Greece.

### 2.4. Statistical Analysis

All analyses were performed in SPSS version 25.0 (IBM SPSS Statistics, Version 25.0), and statistical significance was defined at *p*-value < 0.05 for main effects. Continuous variables were tested for the normality of their distribution using the Kolmogorov–Smirnov test and were treated as means ± standard deviations (SD) in the case of normal distribution or as median/interquartile range (IQR) in the case of non-normal distribution. For those continuous variables that were found not to follow the normal distribution, logarithmic and other transformations took place in an attempt to achieve normality. All categorical variables were presented as numbers (*n*) and percentages (%). Data transformations (i.e., computations and recoding) were also performed, as necessary, to prepare the data for descriptive and inferential statistical analyses.

Descriptive statistical analysis on children’s weight status and EBRB-related variables was conducted for the total sample, as well as by the stratification factors presented in Table S4 for each study. Maternal and paternal descriptive characteristics were also presented as per the available variables. Student’s T-tests and Analysis of Variance (ANOVA) were used to examine the differences between subgroups for normally distributed continuous variables, while the equivalent non-parametric Mann–Whitney and Kruskal–Wallis tests examined these differences in the case of non-normally distributed variables. For post hoc multiple comparisons, the Bonferroni correction was used to account for Type I error. In the case of categorical variables, the chi-square test and (whenever appropriate) Fisher’s exact test for proportions were used to examine associations and differences between subgroups. Associations were examined between (i) children’s weight status and EBRB related variables, and between (ii) EBRB and their determinants.

Subgroup analyses were also performed in order to examine associations between children’s weight status and EBRBs, as well as between EBRBs and their potential determinants, only in children in need as these were identified based on the SES variables summarised in Table S4 for each study. These subgroup analyses allowed the identification of stronger associations in the most vulnerable children that were examined in the present secondary data analyses (i.e., for children of families will lower socioeconomic status, migrant background, etc.).

## 3. Results

### 3.1. Children in Need

Children in need were identified based on available socioeconomic and demographic data, including parental educational level, ethnicity, employment status, region of residence, family income, and family status (i.e., single or dual parent).

According to the results coming from the GENESIS study on toddlers and preschool children (1 to 5 years old), from the total study population, 10.4% and 15.8% were children whose mother and father, respectively, had less than 9 years of education (i.e., the years of compulsory education in Greece), 10.2% were children of economic immigrants, and 20.6% were children living in rural areas and small towns. The ToyBox study was conducted with preschool children (3.5 to 5.5 years old) living in Greece and reported quite similar results. In this regard, from the total ToyBox study population, 25.1% and 24.2% were children whose mother and father, respectively, had less than 12 years of education, 14.5% and 11.4% were those whose mother and father, respectively, were economic immigrants, while 33.3% and 14.3% were children whose mother and father, respectively, were unemployed.

In school children aged 6 to 10 years that participated in the Feel4Diabetes study, 5.4% and 11.7% of the total study sample were those whose mother and father, respectively, had less than 9 years of education, while 41.2% and 26.1% were those whose mother and father, respectively, were unemployed.

Regarding older children of 10 to 12 years old, the Healthy Growth Study showed that 22.2% and 26.4% were those whose mother and father, respectively, had up to 9 years of education, 22.6% had an annual family income of less than 12,000 Euros, 18.4% were those living in rural areas, 16.6% and 15.4% were those whose mother and father, respectively, were non-Greeks (mainly referring to economic immigrants), 10.1% were those belonging to single-parent families, while 32.5% were those with at least one parent that was unemployed. The Energy study, which was also conducted with 10–12-year-old children, reported similar findings to the Healthy Growth with regard to the prevalence of children in need in Greece. In this context, the analysis of the Energy study data showed that from the total study population, 23.1% and 25.9% were children whose mother and father, respectively, had less than 12 years of education, 31.1% were those whose parents were non-Greeks, 9.7% were children from single-parent families, and 36.4% were children where at least one parent was unemployed. Table 2 summarises the prevalence of children in need in different ages groups, based on different socioeconomic and demographic indices, available in each study.

### 3.2. Prevalence of Overweight and Obesity

The prevalence of overweight and obesity in children in need was reported to be equal and, on several occasions, higher than the one reported for the general population of children from different age groups living in Greece. According to the results on toddlers and preschool children from the Genesis study, the prevalence of overweight and obesity was found to reach 17% in children of mothers with less than 9 years of education, 22.9% for children of immigrant parents, and 18.3% for children living in rural areas. Similar percentages were also reported for preschool children participating in the ToyBox study, since the prevalence of overweight and obesity was found to reach 19.6% in children whose father’s education was less than 12 years, 17.5% in children with an immigrant father, and 21.5% in children whose father was unemployed.

The results from older school-aged children participating in the Feel4Diabetes study showed much higher prevalence of overweight and obesity compared to the ones reported for younger children. In this regard, the prevalence of overweight and obesity was found to go up to 64.9% in children whose mother had up to 9 years of education and 42.4% for those whose father was unemployed.

The data analysis of the Healthy Growth and the Energy studies showed quite similar results with regard to the prevalence of overweight and obesity in children in need. More specifically, in the Healthy Growth study the prevalence of overweight and obesity was 44.2% for children whose father had less than 9 years of education, approximately 36% for children with immigrant parents, 44.9% for children living in rural areas, 39.4% for children with unemployed parents, 42.9% for children living in single-parent families, and 39% in children from low-income families. In the Energy study, the prevalence of overweight and obesity was 42.5% in those children whose father had less than 12 years of education, 17% in children of immigrant parents, 60.1% in children whose one or both parents were unemployed, and 42.1% in children from single-parent families. Table 3 presents the prevalence of overweight and obesity in children ‘in need’ from different ages groups, as these were identified based on different socioeconomic and demographic characteristics, available in each study.

### 3.3. Food-Related EBRBs

The EBRBs examined in this secondary data analysis included the consumption of foods and beverages from different food groups and the level of engagement in physical activity and sedentary behaviours.

#### 3.3.1. Genesis Study

In the Genesis study, toddlers and preschool children whose mother had less than 9 years of education were found to consume fewer daily servings of vegetables and fruit (1.8 vs. 2.4 servings per day) compared to children of mothers with more than 12 years of education. Furthermore, toddlers and preschool children living in rural areas were found to have higher daily caloric intake (1484 vs. 1355 kcal per day; *p* < 0.001) but, at the same time, consumed more daily servings of vegetables and fruit (2.6 vs. 2.1 servings per day; *p* < 0.001) compared to children living in urban areas.

#### 3.3.2. ToyBox Study

In the ToyBox study, higher percentages of preschool children whose parents were non-Greek (mainly immigrants) were found to report more frequent consumption of sweet snacks (7.2% vs. 4.2% at least once per day; *p* = 0.028), savoury snacks (6.7% vs. 1.7% at least once per day; *p* < 0.001), sweetened/flavoured milk (17% vs. 10.8% at least once per day; *p* < 0.001), fruit juice (17% vs. 8.7% at least once per day; *p* < 0.001), and sugar-sweetened beverages (11.9% vs. 7.5% at least once per day; *p* < 0.05), compared to children whose parents were Greek nationals. On the contrary, lower percentages of preschool children with non-Greek parents were found to report more frequent consumption of vegetables and fruit (69.1% vs. 75.9% at least once per day; *p* < 0.05) and plain/unsweetened milk (85.1% vs. 92.2% at least once per day; *p* < 0.001) compared to children with Greek parents.

The statistical analysis of the ToyBox study data also revealed higher percentages of children whose mother or father had less than 12 years of education reporting more frequent consumption of savoury snacks (3.1% vs. 2.3% at least once per day; *p* < 0.001), sweetened/flavoured milk (14.8% vs. 10.2% at least once per day; *p* = 0.004), fruit juice (13.7% vs. 8.4% at least once per day; *p* < 0.001), and sugar-sweetened beverages (12.3% vs. 6.5% at least once per day; *p* < 0.001). However, lower percentages of preschool children with parents that had less than 12 years of education were found to report more frequent consumption of vegetables and fruit (66.1% vs. 78.1% at least once per day; *p* < 0.05), plain/unsweetened milk (88.3% vs. 92.5% at least once per day; *p* < 0.001), and water (92.8% vs. 95.9% at least once per day; *p* = 0.011), compared to children of parents with more than 12 years of education.

Employment status of parents was also important since the analyses showed higher percentages of preschool children with more frequent consumption of sweetened/flavoured milk (17.6% vs. 10.3% at least once per day; *p* < 0.001), fruit juice (12.5 vs. 8% at least once per day; *p* = 0.006), and sugar-sweetened beverages (10.6% vs. 6.8% at least once per day; *p* = 0.019) in those with unemployed parents compared to those with both parents employed. On the contrary, lower percentages of preschool children with unemployed parents were found to report more frequent consumption of vegetables and fruit (65.9% vs. 77.4% at least once per day; *p* < 0.001) and plain/unsweetened milk (84.3% vs. 92.8% at least once per day; *p* < 0.001) compared to children whose parents were both employed.

#### 3.3.3. Feel4Diabetes Study

In the Feel4Diabetes study, children whose mother or father had less than 9 years of education were found to consume more servings of full-fat dairy products (2.7 vs. 1.9 servings per day; *p* = 0.006), plain/non-whole-grain cereals (2.6 vs. 1.9 servings per day; *p* = 0.003), soft drinks (1 vs. 0.4 servings per day; *p* < 0.001), and fruit juice with sugar (1.7 vs. 1.1 servings per day; *p* < 0.001) compared to children whose parents had more than 12 years of education. Furthermore, statistical analyses revealed higher percentages of children whose mother or father had less than 9 years of education reporting more frequent consumption of soft drinks with (21.6% vs. 15.2% at least once per day; *p* = 0.009) or without sugar (33.8% vs. 18.6% at least once per day; *p* = 0.012), compared to children whose parents had more than 12 years of education.

On the other hand, children whose mother or father had less than 9 years of education reported lower consumption of whole-grain cereals (0.2 vs. 0.5 servings per day; *p* = 0.003) compared to children whose parents had more than 12 years of education. In addition, lower percentages of preschool children with parents that had less than 9 years of education were found to report more frequent consumption of fresh fruit (35.1% vs. 51.7% at least once per day; *p* = 0.002), vegetables (24.3% vs. 34.4% at least once per day; *p* < 0.001), dairy products (28.6% vs. 53.2% at least once per day; *p* < 0.001)), whole-grain cereals (4.6% vs. 10.3% at least once per day; *p* = 0.001), and breakfast on weekdays (67.1% vs. 86.3%; *p* < 0.001), compared to their counterparts whose parents had more than 12 years of education.

In addition, the analysis showed that higher percentages of children with unemployed parents had higher consumption of water (65.6% vs. 54.8% at least 5 servings per day; *p* < 0.001), fruit juice (32.9% vs. 20.2% at least once per day; *p* < 0.05), vegetables (37.4% vs. 24.7% at least once per day; *p* < 0.001), soft drinks with (22.8% vs. 9.4% at least once per day; *p* < 0.001) or without sugar (30.2% vs. 14.8% at least once per day; *p* < 0.001), and sweet (32% vs. 20.4% at least once per day; *p* < 0.001) and savoury snacks (17.2% vs. 5.4% at least once per day; *p* < 0.001) compared to children whose parents were employed full-time.

#### 3.3.4. Healthy Growth Study

In the Healthy Growth study, children whose parents were non-Greek nationals reported higher caloric intake (1836 vs. 1778 Kcal per day; *p* = 0.044) compared to children whose parents were Greek nationals. Similarly, children from single-parent families were also found to have higher caloric intake compared to children with dual-parent families (1873 vs. 1770 Kcal per day; *p* = 0.008). Regarding food intake, the analysis showed higher percentages in children with parents who have less than 9 years of education reporting more frequent consumption of fruit juice (19.2% vs. 12.5% at least once per day; *p* < 0.001) and soft drinks with sugar (11.3% vs. 4.4% at least once per day; *p* < 0.001), chocolate milk (13.7% vs. 8.9% at least once per day; *p* = 0.006), chocolates (15.8% vs. 9.7% at least once per day; *p* = 0.002), and chips (7.3% vs. 1.5% at least once per day; *p* < 0.001), compared to children with parents that had more than 12 years of education.

However, the analysis also revealed lower percentages of children with parents who have less than 9 years of education reporting more frequent consumption of fruit (33.8% vs. 48.3% at least once per day; *p* < 0.001), vegetables (28.4% vs. 39.1% at least once per day; *p* < 0.001), fresh fruit juice (15.6% vs. 22.3% at least once per day; *p* = 0.009), and cereals (19.6% vs. 28.2% at least once per day; *p* < 0.001), compared to more educated parents. Furthermore, higher percentages of children with non-Greek parents had more frequent consumption of fruit juice (31% vs. 12.7% at least once per day; *p* < 0.001) and soft drinks with sugar (10.9% vs. 5.1% at least once per day; *p* < 0.001) and chocolates (19% vs. 9.9% at least once per day; *p* < 0.001), compared to children with Greek parents, while lower percentages of children with non-Greek parents had more frequent consumption of milk (60.8% vs. 70.3% at least once per day; *p* < 0.001) compared to their Greek counterparts.

Lower percentages of children living in rural areas were found to have more frequent consumption of fruit (32.9% vs. 47%; *p* < 0.001), fresh fruit juice (14.2% vs. 19.9% at least once per day; *p* = 0.002), vegetables (26.1% vs. 39.4% at least once per day; *p* < 0.001), cereals (16.9% vs. 28.9% at least once per day; *p* < 0.001), milk (63.5% vs. 74.2% at least once per day; *p* < 0.001), chocolates (8.4% vs. 12.6% at least once per day; *p* < 0.001), and chips (5.7% vs. 2.4% at least once per day; *p* < 0.001), compared to children living in metropolitan areas. More children from single-parent families were also found to have more frequent consumption of soft drinks with sugar (9.7% vs. 5.4% at least once per day; *p* = 0.026) and chips (5.8% vs. 2.7% at least once per day; *p* = 0.031), compared to those from dual-parent families. The analysis also revealed higher percentages of children from low-income families with a more frequent consumption of fruit juice (21.8% vs. 12.1% at least once per day; *p* < 0.001) and soft drinks with sugar (11.2% vs. 3.5% at least once per day; *p* < 0.001), chocolate milk (13.9% vs. 9.2% at least once per day; *p* = 0.005), chocolates (17.5% vs. 7% at least once per day; *p* < 0.001), and chips (6.6% vs. 1.9% at least once per day; *p* < 0.001), compared to children from higher-income families. On the contrary, lower percentages of children from low-income families had more frequent consumption of fresh fruit juice (17.9% vs. 24.3% at least once per day; *p* < 0.001), vegetables (28.6% vs. 36% at least once per day; *p* = 0.023), and milk (61.8% vs. 75.8% at least once per day; *p* < 0.001), compared to their peers from higher-income families.

#### 3.3.5. Energy Study

In the Energy study, those children whose mother or father had less than 12 years of education had higher consumption of soft drinks (135 vs. 88 mL per day; *p* < 0.001) but less frequent consumption of breakfast (5 vs. 5.7 days per week; *p* < 0.001), compared to children whose parents had more than 14 years of education. Children of parents that were not Greek nationals reported higher consumption of soft (144.6 vs. 90 mL per day; *p* < 0.001) and other sugared-sweetened drinks (412.6 vs. 338.4 mL per day; *p* = 0.002), but less frequent consumption of breakfast (5.2 vs. 5.6 days per week; *p* = 0.012) compared to children of Greek parents. Furthermore, children from single-parent families were also found to have less frequent breakfast consumption compared to children from dual-parent families (4.8 vs. 5.5 days per week; *p* < 0.001).

### 3.4. Physical Activity and Sedentary Behaviour-Related EBRBs

#### 3.4.1. Genesis Study

In the Genesis study, toddlers and preschool children whose mother had less than 9 years of education were found to spend more time on screen activities (1.8 vs. 1.6 h per day; *p* < 0.001) compared to children of mothers with more than 12 years of education. Furthermore, children of immigrant parents were found to spend an average of 2.3 h per day on screen-related activities (i.e., TV watching, video games, etc.), which was higher compared to the 1.7 h per day spent on screen activities by children of Greek parents (*p* < 0.001). Furthermore, toddlers and preschool children living in rural areas were found to spend more time on moderate-to-vigorous physical activities (3.3 vs. 0.6 h per week; *p* < 0.001) but less time on screen activities (1.3 vs. 1.8 hrs per day; *p* < 0.001) compared to children living in urban areas.

#### 3.4.2. ToyBox Study

A higher percentage of preschool children whose parents were non-Greeks were found to engage for more than 2 h per day in screen activities during weekend days, compared to children whose parents were Greek nationals (44.3% vs. 36.7%; *p* < 0.05). In addition, the percentage of preschool children who were more physically active, by devoting more than 2 h per day in quiet play, was found to be lower in children of non-Greek/immigrant parents compared to those of Greek parents (16% vs. 16.8%; *p* = 0.011). Regarding energy expenditure-related behaviours, a higher percentage of preschool children whose parents had up to 12 years of education were found to spend more than 2 h per day on screen activities during the weekdays (20.1% vs. 11.8%; *p* < 0.001) and weekend days (45.5% vs. 35.1%; *p* < 0.001) compared to those whose parents had more than 12 years of education. Employment status of parents was also important since the analyses showed higher percentages of preschool children with more than 2 h of screen activities both during the weekdays (19.3% vs. 10.4%; *p* < 0.001) and weekend days (40.5% vs. 36.8%; *p* = 0.019) in those with unemployed parents compared to those whose parents were both employed.

#### 3.4.3. Feel4Diabetes Study

Regarding parental employment status, children with an unemployed father had less sleep time during weekdays compared to those with both parents working full-time (8.5 vs. 8.7 h per day; *p* = 0.002). Nevertheless, a lower percentage of children with unemployed parents were found to have at least 60 min of physical activity during all weekdays compared to children with full-time employed parents (39.5% vs. 46.5%; *p* = 0.005).

#### 3.4.4. Healthy Growth Study

In the Healthy Growth study, school children (10 to 12 years old) whose mother or father had less than 9 years of education reported spending more time on screen activities (3.2 vs. 2.8 h per day; *p* < 0.001), but less time on organised moderate-to-vigorous physical activities (18 vs. 27 min per day; *p* < 0.001) compared to children whose parents had more than 12 years of education. In the Healthy Growth study, children whose parents were non-Greek nationals reported more screen time (3.5 vs. 2.9 h per day; *p* < 0.001) compared to children whose parents were Greek nationals. Furthermore, children from families with a lower annual income were also found to spend more time on screen activities (3.4 vs. 2.7 h per day; *p* < 0.001), but less time on organised moderate-to-vigorous physical activities (19 vs. 30 min per day; *p* < 0.001) compared to children from families with a higher annual income.

#### 3.4.5. Energy Study

In the Energy study, those school children (10 to 12 years old) whose mother or father had less than 12 years of education had higher screen time (131 vs. 116 min per day; *p* = 0.004) and less time devoted to sports participation and active transportation (176 vs. 223 min per week; *p* < 0.001), compared to children whose parents had more than 14 years of education. Children of parents that were not Greek nationals reported higher screen time (209.5 vs. 188.8), but less time spent on sports participation and active transportation (181.6 vs. 212.3 min per day; *p* = 0.004) compared to children of Greek parents.

Table 4 summarises those EBRBs that were found to be higher or lower in children in need from different age groups, as these were identified based on different socioeconomic and demographic characteristics, compared to the rest of the children that participated in each one of the five studies. 

### 3.5. EBRBs Associated with Obesity

EBRBs positively or negatively associated with obesity in children in need are summarised in Table 5. Specifically, observed associations in preschool children participating in the Toybox study were only positive with daily screen time and consumption of sweet snacks in children whose parents had less than 12 years of education, were not Greek nationals, and were unemployed.

In school-aged children participating in the Feel4Diabetes study, obesity was found to be positively associated with daily soft drink consumption without sugar, as well as with more than 2 h of screen time per day for those children whose parents had less than 9 years of education or were unemployed. On the contrary, in those children with less-educated (<9 years) or unemployed parents, obesity was negatively associated with daily consumption of full-fat and unsweetened dairy products. In addition, in those children whose parents were unemployed, obesity was also negatively associated with daily breakfast consumption, sleep time, and more than 60 min of moderate-to-vigorous physical activity.

Regarding older school children participating in the Healthy Growth study, obesity was found to be positively associated with weekly fast-food consumption in those children whose parents were non-Greek nationals and/or were unemployed and with screen time in children from rural areas from single-parent and low-income families. More frequent consumption of pasta and French fries were also found to be associated with obesity in children from single-parent and low-income families, respectively. However, obesity was negatively associated with daily step count in those children whose parents were less educated (<9 years), non-Greek nationals, and unemployed and for children living in rural areas as well as for children of single-parent and low-income families. Daily milk consumption was also found to be negatively associated with obesity in children with less-educated parents.

Finally, in older school children participating in the Energy study, there were only negative associations observed between specific EBRBs and child obesity. In this regard, child obesity was found to be negatively associated with daily breakfast consumption in those children whose parents had less than 12 years of education, were non-Greek nationals, and/or were unemployed, while the duration of daily sleep time was also found to be negatively associated with obesity in children whose parents were less educated and/or were unemployed.

### 3.6. Determinants of EBRBs Associated with Obesity

The EBRBs found to be positively associated with child obesity also guided the selection of the most relevant independent variables examined for their role as potential determinants of these EBRBs. For instance, screen time, which was one of the EBRBs that was consistently found to be positively associated with child obesity in almost all studies, was associated with variables that reflect children’s personal preferences, parental practices, as well as other factors from children’s social and physical environment that were relevant to children’s screen time habits. Table 6 summarises the determinants of EBRBs that were found to be significantly associated with obesity in children in need from different age groups. Table 7 presents the determinants of EBRBs related to components of the socio-ecological model.

More specifically, in preschool children that participated in the ToyBox study the main factors that were found to be positively associated with sweet snack consumption included availability of sweet snacks to children, absence of rules and/or limitations in children’s sweet snack consumption, and parents who do not perceive snacks as bad for their child. On the contrary, when parents restrict their children from snacking while watching TV, permit sweet snack consumption only on certain occasions (e.g., birthday), restrain from eating sweet snacks in front of their children, and do not reward their children with sweet snacks, consumption of sweet snacks from their child is much lower/less frequent.

In school-aged children that participated in the Feel4Diabetes study, the only determinant of soft drinks consumption was their increased availability at home. Regarding the time school children engage in screen activities, this was higher when parents watch TV with their children frequently, when there are no rules limiting screen time, and when there is a TV or another screen/device (i.e., game console, tablet/iPad, smartphone) in the child’s room. Furthermore, children were more physically active when their parents were also physically active with them, when they rewarded their children for being physically active, and when they provided them with extra support for being physically active by taking their children to playgrounds, parks, etc. Regarding children’s sleep time, the presence of a TV or another screen/device in the child’s room was negatively associated with the child’s daily sleep duration.

In older school children that participated in the Healthy Growth Study, food insecurity (specifically when parents believe that there is not enough food available for their family) was associated with lower milk consumption by children. Furthermore, high availability of French fries at home was positively associated with higher consumption of French fries by children. Regarding children’s screen time, this was found to be positively associated with the presence of a TV or another screen/device (i.e., game console, tablet/iPad, smartphone) in the child’s room and with internet availability/access at home. However, when parents limit their child’s screen time, this also reduces the time their child engages in screen activities. Concerning physical activity levels, children’s step count was found to be higher when there are parks, playgrounds, school yards, pedestrian zones, and sport centres that do not require paid registration in the neighbourhood where the child lives. Higher step count was also observed in children that live in neighbourhoods that are considered safe. On the contrary, for those living in neighbourhoods with more criminal incidents and where parents believe that it is safer for children to stay at home rather than spending time outdoors, children’s step count was found to be low.

Regarding older school children that participated in the Energy study, daily breakfast consumption was higher in children who believe that eating breakfast is good, who like eating and find it easy to eat breakfast, who believe that this behaviour is good by their parents and/or friends, who eat breakfast at a set table at home with their parents, whose parents encourage them to eat breakfast and pay attention to what their child will have for breakfast, and when breakfast products (i.e., milk, cereals, etc.) are available at home. However, when children believe that eating breakfast will make them fat, when their parents permit them to skip breakfast and when there is an absence of rules related to breakfast consumption at home, this leads to much lower/less frequent breakfast consumption by children.

## 4. Discussion

The current work analysed data from five large-scale epidemiological studies conducted in Greece between 2004 and 2018 to identify children in need and examine the most significant EBRBs associated with obesity, as well as the key determinants of these behaviours.

According to the WHO, Greece has some of the highest rates of childhood obesity in Europe with particular concern surrounds vulnerable children, especially those living in regions with lower SES, where the rates of childhood obesity tend to be even higher [8]. The prevalence of overweight and obesity among children who fall under the category of children in need, as identified in this analysis, is comparable to or exceeds that observed in the general child population in Greece, highlighting the urgent need for targeted interventions. Depending on the SES and demographic indicators applied, approximately 5% to 41% of 1- to 12-year-old children in Greece fall under the category of children in need.

### 4.1. EBRBs and Their Determinants

The most important EBRBs associated with a higher prevalence of obesity among children in need included frequent consumption of sweet snacks, soft drinks, fast food, French fries, and pasta, as well as increased time spent on recreational screen activities (e.g., television, video games, tablets/iPads, smartphones). These findings are consistent with the existing literature, which indicates that high consumption of added sugars, regardless of overall dietary quality, is strongly linked to an increased risk of overweight and obesity in children [26]. The WHO also reports that higher intake of sugar-sweetened beverages is associated with an increased risk of overweight and obesity in childhood, recommending that free sugars comprise less than 5% of total daily energy intake [27]. Regarding screen time, several studies have identified a positive association between excessive screen use and increased risk of overweight and obesity in children, particularly during adolescence [28,29,30].

In contrast, several protective EBRBs were found to be negatively associated with obesity among children in need. These included daily consumption of plain or unsweetened milk and yoghurt, regular breakfast consumption, adequate sleep duration, and increased time spent in moderate-to-vigorous physical activities. A recent systematic review and meta-analysis confirmed that children and adolescents who skip breakfast are at increased risk of developing overweight or obesity, supporting the findings of this analysis which identified a negative association between daily breakfast consumption and obesity [31]. The protective role of physical activity in reducing obesity risk is well-established; however, only around 20% of youth meet the WHO’s recommendation of at least 60 min of moderate-to-vigorous physical activity per day [32], further emphasising the importance of promoting physical activity in this population. Sleep duration has also been consistently linked to obesity-related outcomes, with evidence showing that insufficient sleep is associated with obesogenic behaviours and increased obesity prevalence in both children and adults [33]. These findings highlight the need for targeted health promotion programmes in Greece that prioritise the reduction of high-risk EBRBs while encouraging the adoption of protective behaviours.

Understanding the underlying determinants of these EBRBs is essential for the development of effective obesity prevention programmes. Findings from this secondary analysis indicate that the majority of determinants influencing both risk and protective EBRBs are rooted in interpersonal factors. These include parental beliefs and attitudes regarding their children’s dietary intake and screen time, parental role modelling, the presence or absence of family rules related to snacking and screen use, and the availability of snacks and screen devices in the household. Supporting this, a study examining parental influences on childhood obesity in low-SES populations identified several contributing factors, such as parental education, occupational status, parenting styles, and feeding practices [34]. The authors emphasised that effective obesity interventions targeting dietary behaviour change should engage not only children but also their parents, particularly those living in socioeconomically disadvantaged communities [34].

In addition to interpersonal factors, individual determinants of EBRBs were also identified, with the most prominent being children’s personal preferences and perceived social norms. A recent shift in obesity research has encouraged the application of a social influence approach, recognising the role of peer and family dynamics in shaping children’s eating behaviours. This is particularly relevant given the tendency of children to use food as a means of fitting in both at home and in school settings [35]. Furthermore, the analysis revealed community-level determinants, particularly aspects of the physical environment and neighbourhood safety, which influence children’s opportunities for physical activity. The importance of the physical environment is well-supported in the literature, with studies indicating that access to green space, parks, and recreational facilities may have a protective effect against obesity by promoting physical activity among children [36].

### 4.2. Current and Future Interventions

The National Action Against Childhood Obesity (2022–2026) programme, launched by the Ministry of Health in collaboration with UNICEF Greece, aims to reduce childhood obesity rates to 24.5% by 2030. This multisectoral initiative brings together UNICEF, the Ministries of Health and Education, and academic partners to promote healthier lifestyles through education, improved access to nutritious food, and increased opportunities for physical activity in schools and communities [7]. The findings of this secondary data analysis align with and support the objectives of the programme by highlighting the critical role of EBRBs (including diet, physical activity, and sedentary behaviour) and their underlying determinants in shaping the risk of childhood obesity. The findings can be generalised to both the vulnerable and invulnerable populations of children across all countries that prioritise preventing childhood obesity.

Regarding future interventions aimed at preventing and reducing childhood obesity among children from low socioeconomic backgrounds, the findings of this analysis highlight the importance of targeting both protective and high-risk EBRBs. The school environment plays a critical role in shaping children’s dietary choices and physical activity levels [37]. As such, prioritising the development of health-promoting school environments is essential. Interventions should focus on creating supportive settings that facilitate access to nutritious foods, encourage regular physical activity, and reduce sedentary behaviours. These approaches are in line with the goals of national initiatives such as the National Action Against Childhood Obesity, which emphasise school and community-based strategies to promote healthier lifestyles among children [7].

The influence of socioeconomic status (SES) on obesity risk has been well-documented, with numerous studies demonstrating a clear association between low SES, both at the household and neighbourhood levels, and increased rates of childhood obesity [13,38]. Given the increased vulnerability of children from low-SES backgrounds, it is imperative that obesity prevention efforts prioritise this population. Strengthening preventative strategies tailored to their specific needs can play a critical role in reducing the burden of obesity and mitigating its long-term health and social consequences.

### 4.3. Strengths and Limitations

It is important to acknowledge the strengths and limitations of this secondary data analysis to fully understand its potential impact on the validity and applicability of the findings. A key strength of the analysis lies in the inclusion of five large-scale epidemiological studies, each with substantial sample sizes and the use of randomised sampling methods, thereby enhancing the representativeness and generalisability of the results. Additionally, the analysis covered a broad age range of children (1–12 years), which allowed for comparisons across different developmental stages; for example, identifying differences in significant EBRBs between younger children (1–5 years) and older children (10–12 years). This age-stratified approach enabled the identification of age-specific risk factors and protective behaviours related to obesity. Another strength pertains to the data collection methods used across the studies included in this analysis. The use of official documents, such as birth certificates and health records to obtain key variables data, helped minimise recall bias for those data points. Moreover, data collection, particularly anthropometric measurements, was conducted by trained researchers using standardised procedures, thus improving the accuracy and reliability of the data.

However, several limitations must also be considered. One key limitation is the retrospective nature of the data, which restricts the ability to infer causality and allows only for the identification of associations. While some data were objectively collected (e.g., anthropometry and perinatal health records), other variables relied on parental reports. The use of parental recall introduces potential bias, especially when mothers are asked to report on events or behaviours that occurred years prior to the data collection period. This may compromise the accuracy of certain variables and reduce the overall strength of some findings. Furthermore, insufficient data and heterogeneity made it unfeasible to perform a meta-analysis.

## 5. Conclusions

The prevention of childhood obesity in Greece, particularly among vulnerable children from lower socioeconomic backgrounds, remains a critical public health priority. This analysis identified several EBRBs that either increase (risk EBRBs) or reduce (protective EBRBs) the likelihood of obesity in this population, along with key interpersonal, individual, and environmental determinants influencing these behaviours. Future public health policies and interventions should prioritise addressing these modifiable EBRBs to reduce the risk of obesity and, in turn, alleviate the associated health burden on children, both vulnerable or not, their families, and broader communities.

## Figures and Tables

**Table 1:** Characteristics of studies included in the secondary data analysis.

Study Name	Age Range (Years)	Population	Sample Size	Year(s)	Duration	Study Sites (Greece)
Genesis	1–5	Nurseries and day-care centres	2374	2003–2004	15 months	Attica, Thessaloniki, Etoloakarnania, Halkidiki
ToyBox	3.5–5.5	Kindergartens	1229	2010–2014	4 years	Attica
Feel4Diabetes	6–10	Primary schools (Grades 1–3)	2286	2016–2018	2 years	Attica
Healthy Growth	10–12	Primary schools (Grades 5–6)	2294	2007–2009	25 months	Attica, Thessaloniki, Etoloakarnania, Iraklio Crete
Energy	10–12	Primary schools (Grades 5–6)	1077	2010	4 months	Attica

**Table 2:** Percentage of children in need in different age groups for the five studies.

Study Name	Educational Level (Years)		Non-Greek Ethnicity (Immigrants)	Rural Area	Unemployed Parents	Single- Parent Families	Low Family Income *
	<9	<12					
Genesis (1–5 years old)	10.4% (M) 15.8% (F)		10.2%	20.6%			
ToyBox (3.5–5.5 years old)		25.1% (M) 24.2% (F)	14.5% (M) 11.5% (F)		33.3% (M) 14.3% (F)		
Feel4Diabetes (6–10 years old)	5.4% (M) 11.7% (F)				41.2% (M) 26.6% (F)		
Healthy Growth (10–12 years old)	22.6% (M) 26.4% (F)		16.6% (M) 15.4% (F)	18.4%	32.5%	10.1%	22.6%
Energy (10–12 years old)		23.1% (M) 25.9% (F)	31.1%		36.4%	9.7%	

M: % of children in need based on mother’s educational level or employment status; F: % of children in need based on father’s educational level. *: Low income is defined as less than 12,000 Euros per year.

**Table 3:** Prevalence of overweight and obesity in children in need from different age groups for the five studies.

Study Name	Educational Level (Years)		Non-Greek Ethnicity (Immigrants)	Rural Area	Unemployed Parents	Single- Parent Families	Low Family Income *
	<9	<12					
Genesis (1–5 years old)	10.9% (Ov) 6.1% (Ob) (M) 10.9% (Ov) 5.7% (Ob) (F)		14.9% (Ov) 8% (Ob)	10.7% (Ov) 7.6% (Ob)			
ToyBox (3.5–5.5 years old)		13.9% (Ov) 5.1% (Ob) (M) 14.8% (Ov) 4.8% (Ob) (F)	12.3% (Ov) 3.3% (Ob) (M) 12.9% (Ov) 4.6% (Ob) (F)		15.5% (Ov) 5.6% (Ob) (M) 13.7% (Ov) 7.8% (Ob) (F)		
Feel4Diabetes (6–12 years old)	31.1% (Ov) 33.8% (Ob) (M) 19.8% (Ov) 16.7% (Ob) (F)				23.9% (Ov) 16.1% (Ob) (M) 25.4% (Ov) 17% (Ob) (F)		
Healthy Growth (10–12 years old)	29.3% (Ov) 14.9% (Ob) (M) 26.4% (Ov) 14.7% (Ob) (F)		25.5% (Ov) 10.7% (Ob) (M) 25.7% (Ov) 10.3% (Ob) (F)	31.5% (Ov) 13.4% (Ob)	26% (Ov) 13.4% (Ob)	31.1% (Ov) 11.8% (Ob)	27.5% (Ov) 11.5% (Ob)
Energy (10–12 years old)		26% (Ov) 12.6% (Ob) (M) 28.5% (Ov) 14% (Ob) (F)	25% (Ov) 12% (Ob)		50.7% (Ov) 9.4% (Ob)	32.6% (Ov) 9.5% (Ob)	

Ov: overweight; Ob: obesity; M: % of Ov or Ob based on mother’s educational level; F: % of Ov or Ob based on father’s educational level. *: Low income is defined as less than 12.000 Euros per year.

**Table 4:** Most prevalent energy balance-related behaviours in children in need from different age groups for the five studies.

Study Name	Educational Level (Years)		Non-Greek Ethnicity (Immigrants)	Rural Area	Unemployed Parents	Single-Parent Families	Low Family Income *
	<9	<12					
Genesis (1–5 years old)	Daily vegetables (↓) Daily fruit (↓) Screen time (↑)		Screen time (↑)	Dietary energy intake (↑) Daily vegetables (↑) Daily fruit (↑) MVPA (↑) Screen time (↓)			
ToyBox (3.5–5.5 years old)		Daily savoury snacks (↑) Daily sweetened/ flavoured milk (↑) Daily fruit juice (↑) Daily sugar-sweetened beverages (↑) Screen time (↑) Daily vegetables (↓) Daily fruit (↓) Daily plain/unsweetened milk (↓) Daily water (↓)	Daily sweet snacks (↑) Daily savoury snacks (↑) Daily sweetened/flavoured milk (↑) Daily sugar-sweetened beverages (↑) Screen time (↑) Daily vegetables (↓) Daily fruit (↓) Daily plain/unsweetened milk (↓) Daily quiet play time (↓)		Daily sweetened/ flavoured milk (↑) Daily fruit juice (↑) Daily sugar-sweetened beverages (↑) Screen time (↑) Daily vegetables (↓) Daily fruit (↓) Daily plain/ unsweetened milk (↓)		
Feel4Diabetes (6–10 years old)	Daily full-fat dairy products (↑) Daily plain/refined grain products (↑) Daily sugar-sweetened soft drinks (↑) Daily sugar-sweetened fruit juice (↑) Daily whole-grain cereals (↓) Daily vegetables (↓) Daily fruit (↓) Daily dairy products (↓) Daily breakfast (↓)				Daily water (↑) Daily fruit juice (↑) Daily vegetables (↑) Daily soft drinks with and without sugar (↑) Daily sweet snacks (↑) Daily savoury snacks (↑) Daily sleep time (↓) Daily 60 min of PA (↓)		
Healthy Growth (10–12 years old)	Daily sugar-sweetened fruit juice (↑) Daily sugar-sweetened soft drinks (↑) Daily chocolate milk (↑) Daily chocolates (↑) Daily chips (↑) Screen time (↑) Daily vegetables (↓) Daily fruit (↓) Daily fresh fruit juice (↓) Daily cereals (↓) Organised MVPA (↓)		Dietary energy intake (↑) Daily sugar-sweetened soft drinks (↑) Daily sugar-sweetened fruit juice (↑) Daily chocolates (↑) Screen time (↑) Daily milk (↓)	Daily vegetables (↓) Daily fruit (↓) Daily fresh fruit juice (↓) Daily cereals (↓) Daily milk (↓) Daily chocolates (↓) Daily chips (↓) Organised MVPA (↓)		Dietary energy intake (↑) Daily sugar-sweetened soft drinks (↑) Daily sugar-sweetened fruit juice (↑) Daily chocolate milk (↑) Daily chocolates (↑) Daily chips (↑) Daily fresh fruit juice (↓) Daily vegetables (↓) Daily milk (↓)	Screen time (↑) Organised MVPA (↓)
Energy (10–12 years old)		Daily sugar-sweetened soft drinks (↑) Screen time (↑) Daily breakfast (↓) Daily sports and active transportation (↓)	Daily sugar-sweetened soft drinks (↑) Screen time (↑) Daily breakfast (↓) Daily sports and active transportation (↓)			Daily breakfast (↓)	

*: Low income is defined as less than 12,000 Euros per year. The ↑ and ↓ symbols indicate that the specific EBRB is significantly higher or lower, respectively, in ‘children in need’ compared to children from higher socioeconomic groups (*p* < 0.05). MVPA: moderate-to-vigorous physical activity; PA: physical activity.

**Table 5:** Energy balance-related behaviours found to be associated with obesity in children in need from different age groups for the five studies.

Study Name	EBRBs Positively or Negatively Associated with Obesity in Children in Need
Genesis (1–5 years old)	No significant associations were observed
ToyBox (3.5–5.5 years old)	EBRBS in children with low-educated, non-Greek, and/or unemployed parents: Screen time: Time children engage in screen activities (i.e., TV, video, game consoles, tablets/iPads, smartphones (+) Consumption of sweet snacks (i.e., cakes, biscuits, candy, etc.) (+)
Feel4Diabetes (6–10 years old)	EBRBS in children with low-educated and/or unemployed parents: Soft drinks with and without sugar (+) Screen time: Time children engage in screen activities (i.e., TV, video, game consoles, tablets/iPads, smartphones (+) -------------------------------------------------------------------------------------------------------------------------------- Consumption of full-fat and unsweetened dairy products (i.e., milk and yoghurt) (−) Daily breakfast consumption (−) Sleep duration (−) Time children engage in moderate-to-vigorous physical activities (−)
Healthy Growth (10–12 years old)	EBRBS in children with low-educated and/or non-Greek and/or unemployed parents, children living in rural areas, and parents and children from single-parent families and/or low-income families: Frequent consumption of fast food, French fries, and pasta (+) Screen time: Time children engage in screen activities (i.e., TV, video, game consoles, tablets/iPads, smartphones (+) -------------------------------------------------------------------------------------------------------------------------------- Daily step count (−) Consumption of milk (−)
Energy (10–12 years old)	EBRBS in children with low-educated and/or unemployed parents: Daily breakfast consumption (−) Sleep duration (−)

The (+) and (−) symbols indicate significant positive and negative associations (*p* < 0.05), respectively, between determinates and EBRBs that were found to be significantly associated with obesity (*p* < 0.05) in ‘children in need’.

**Table 6:** Determinants of energy balance-related behaviours associated with obesity in children in need from different age groups.

Study Name	Determinants of EBRBs Associated with Obesity in “Children in Need”.
Genesis (1–5 years old)	
ToyBox (3.5–5.5 years old)	Determinants of sweet snack consumption in children with non-Greek and/or unemployed parents: Parents who make sweet snacks regularly available to their child (+) Parents who find it difficult to limit their child’s sweet snacks consumption (+) Absence of rules in limiting snacks consumption (+) Parents who believe sweet snack consumption is NOT bad for their child (+) -------------------------------------------------------------------------------------------------------------------------------- Parents restricting snacking of their children while watching TV (−) Parents who permit consumption of sweet or salty snacks only in certain occasions (e.g., birthdays) (−) Parents who can restrain themselves from eating sweet snacks in from of their child (−) Parents who DO NOT offer sweet snacks to their child as a reward (−) Determinants of screen time in children with low-educated and/or non-Greek parents: Child’s personal preference in watching TV/DVD/video (+) Parents who find it difficult to limit their child’s screen time (+) Parents watching TV with their children (+) Absence of rules in limiting screen time (+) Parents who believe that screen viewing is beneficial/educational for their child (+) -------------------------------------------------------------------------------------------------------------------------------- Parents who are pleased with their child’s screen time (−) Parents who believe that their child’s screen time is within the recommended levels (−) Parents providing other activities as alternatives to screen time to their children (−) Parents who believe it is necessary to limit their child’s screen time (−)
Feel4Diabetes (6–10 years old)	Determinants of soft drink consumption in children of low-educated and/or unemployed parents: Soft drink availability at home (+) Determinants of screen time in children with low-educated parents: Parents frequently watching TV with their child (+) Absence of rules in limiting screen time (+) Presence of TV, tablet/iPad and/or smartphone in child’s room (+) Determinants of physical activity in children with unemployed parents: Parents who are physically active and exercise with their child (+) Parents who reward their child for being physically active (+) Parents who support their child’s physical activity by taking them to the playground/park (+) Determinants of sleep time in children with unemployed parents: Presence of TV, game console, computer, tablet/iPad, and/or smartphone in child’s room (−)
Healthy Growth (10–12 years old)	Determinants of milk consumption in children with low-educated parents: Parental food insecurity (not enough food availability) (−) Determinants of French fry consumption in children from low-income families: Availability of French fries at home (+) Determinants of screen time in children living in rural areas and those from single-parent families: Presence of TV, Tablet/iPad and/or smartphone in child’s room (+) Internet access at home (+) ------------------------------------------------------------------------------------------------------------------------------- Parents who limit their child’s screen time (−) Determinants of step count/physical activity in children with low-educated and/or non-Greek parents, in children living in rural regions, and in those from single-parent and/or low-income families: Availability of parks, playgrounds, pedestrian zones, school yards, and sport centres with no enrolment fee near home (+) Safety in the neighbourhood for the child to play (+) ------------------------------------------------------------------------------------------------------------------------------- Neighbourhood with more criminal incidents (−) Parents who believe it is safer for the child to stay at home than playing out in the neighbourhood (−)
Energy (10–12 years old)	Determinants of breakfast consumption in children with low-educated, non-Greek, and/or unemployed parents: Children who believe that eating breakfast is good (+) Children who like eating breakfast (+) Children who find eating breakfast easy (+) Children who believe that when eating breakfast their parents and friends think it is good/very good (+) Children who eat their breakfast at a set table at home (+) Breakfast consumption with parents (+) Parents who encourage their child to eat breakfast (+) Parents who pay attention to what their child consumes for breakfast (+) Availability of breakfast food products (i.e., milk, cereals, etc.) at home (+) ------------------------------------------------------------------------------------------------------------------------------- Children who believe that eating breakfast will make them fat (−) Parents who permit their children to skip breakfast (−) Absence of rules related to breakfast consumption (−)

The (+) and (-) symbols indicate positive and negative associations, respectively, between determinates and EBRBs that were found to be associated with obesity in “children in need”.

**Table 7:** Determinants of EBRBs related to components of the socio-ecological model.

Socio-Ecological Model Components
Individual
Determinants that were positively associated with the relevant EBRBs: Child’s personal preference in watching TV/DVD/video. Children who believe that eating breakfast is good. Children who like and find it easy to eat breakfast. Children who believe that when eating breakfast their parents and friends think it is good/very good. Determinants that were negatively associated with the relevant EBRBs: Children who believe that eating breakfast will make them fat.
Interpersonal
Determinants that were positively associated with the relevant EBRBs: Parents who make sweet snacks and/or soft drinks regularly available to their child. Parents who find it difficult to limit their child’s sweet snacks consumption and/or screen time. Absence of rules in limiting snack consumption and/or screen time. Parents who believe sweet snack consumption is NOT bad for their child. Parents who believe that screen viewing is beneficial/educational for their child. Parents who frequently watch TV with their child. Presence of TV, tablet/iPad, and/or smartphone in child’s room. Internet access at home. Children who eat their breakfast at a set table at home. Breakfast consumption with parents. Parents who encourage their child to eat breakfast. Parents who pay attention to what their child consumes for breakfast. Availability of breakfast food products (i.e., milk, cereals, etc.) at home. Determinants that were negatively associated with the relevant EBRBs: Parents restricting snacking of their children while watching TV. Parents who permit consumption of sweet or salty snacks only on certain occasions (e.g., birthdays). Parents who can restrain themselves from eating sweet snacks in from of their child. Parents who DO NOT offer sweet snacks to their child as a reward. Parents who are pleased with their child’s screen time. Parents who believe that their child’s screen time is within the recommended levels. Parents providing other activities as alternatives to screen time to their children. Parents who believe it is necessary to limit their child’s screen time. Presence of TV, game console, computer, tablet/iPad, and/or smartphone in child’s room. Parental food insecurity (not enough food availability). Parents who limit their child’s screen time. Parents who believe it is safer for the child to stay at home than playing out in the neighbourhood. Parents who permit their children to skip breakfast. Absence of rules related to breakfast consumption.
Community
Determinants that were positively associated with the relevant EBRBs: Availability of parks, playgrounds, pedestrian zones, school yards, and sport centres with no enrolment fee near home. Safety in the neighbourhood for the child to play. Determinants that were negatively associated with the relevant EBRBs: Neighbourhood with criminal incidents.

## Data Availability

The data presented in this study are available on request from the corresponding author.

## Supplementary Materials

The following supporting information can be downloaded at: https://www.mdpi.com/article/10.3390/nu17213486/s1, Table S1: Children’s weight status related variables by study; Table S2: Energy balance related behaviour variables by study; Table S3: Potential determinants of EBRBs by study; Table S4: Socio-economic status variables used for the identification of vulnerable populations of “children in need”.
