# Supplementary material for: Associations Between Energy Balance-Related Behaviours and Childhood Obesity Among Vulnerable Populations in Greece: Implications for Public Health Policy and Intervention Development
Source: Nutrients. 2025 Nov 6;17(21):3486. doi: 10.3390/nu17213486 (PMC12610217; doi:10.3390/nu17213486)
Supplement: Supplementary file 1 [file nutrients-17-03486-s001.zip › Supplementary Materials.pdf]

Supplementary Materials

Table S1. Children's weight status related variables by study.

Study	Weight status
Genesis<br>ToyBox<br>Feel4Diabetes<br>Healthy Growth<br>Energy	Continuous variables:<br>Body weight (kg)<br>Height (cm)<br>Body Mass Index (Kg/m^2)<br>BMI z-score*<br>Waist circumference (cm)†
	Categorical variables:<br>BMI status categories**<br>Waist circumference categories***

†: Waist circumference was not measured in the Feel4Diabetes study.

*BMI z-scores is calculated according to the World Health Organization Growth Standards [1,2].

**BMI status categorization was based on the International Obesity Task Force cut-off points

***Waist circumference (WC) categorization was based on age- and sex-specific WC percentile thresholds [3]: Central obesity ≥ 90th Percentile.

Table S2. Energy balance related behaviour variables by study.

Study	Energy balance related behaviours (EBRBs)
Genesis	Continuous variables:<br>Dietary energy intake (Kcal/day).<br>Vegetables, fruits, fish and seafood, whole grains, sugar-sweetened beverages (serves/day)<br>Light-to-vigorous intensity physical activity levels (min/day)<br>Time spent watching TV (hours/day)<br>Screen time other than TV watching (hours/day)<br>Sleep time (hour/day)
	Categorical variables:<br>Eating while watching TV
ToyBox	Continuous variables:<br>TV/Computer/DVD/Game consoles (hours/day on weekdays and weekends)<br>Sleep time (hours/day on weekdays and weekends)
	Categorical variables:<br>The foods listed below are those consumed by children on a daily, weekly, or monthly basis. Respondents need to choose one of the following response choices regarding the child's food intake: Never; 1 day per week; 2-4 days per week; 5-6 days per week; 1-2 days per day; 3-4 times per day; 5 or more times per day.<br>Breakfast; Water; Fruit Juice (home-made or packaged); Light sodas; sugar-sweetened beverages; Sweets/candies/chocolate; Biscuits/cookies/cakes/muffins; Crisps and other similar salty snacks; Fruit and vegetables; Pizza, cheese pies/ meat pies; Milk (plain); Yogurt (plain); Milk (flavoured); Yogurt (flavoured); Cheese.
	Continuous variables:

Feel4Diabetes	Dairy low-fat serving (120mL).<br>Dairy full fat serving (120mL).<br>Child Cereals wholegrain bread serving 30g.<br>Child Cereals non whole grain bread serving 30g.<br>Child Cereals porridge serving 30g.<br>Child Cereals wholegrain cereals serving 30g.<br>Child Cereals non whole grain cereals serving 30g.<br>Child Organised Activity 1 times per week.<br>Child Organized Activity 1 min per day.<br>Child Sleep hours weekdays/night<br>Child Sleep hours weekend days<br>Legumes times per week.<br>Child Red meat times per week<br>Child White meat times per week<br>Child Fish times per week<br>Child Water serving 250mL<br>Child Tea serving 250mL<br>Sugar per cup<br>Sweetener per cup<br>Soft drinks with sugar serving 250mL<br>Soft drinks without sugar serving 250mL<br>Juice without sugar serving 250mL<br>Juice with sugar serving 250 mL
	Categorical variables:<br>The foods listed below are those consumed by children on a daily, weekly, or monthly basis. Respondents need to choose one of the following response choices regarding the child's food intake: 0 time per week; 1 or 2 times per week; 3 or 4 times per week; 5 or 6 times per week; 1 or 2 times per day; 3 or 4 times per day; 5 or 6 times per day; > 6 times per day)<br>Water (1 glass or 1 cup)<br>Fruits and berries, fresh or frozen (1 tennis ball sized fruit or 2 small fruits or half a cup of chopped)<br>Fruits and berries, canned (half a cup) or dried (a quarter of cup)<br>Fruit juices, freshly squeezed or prepacked without sugar (1 glass or 1 cup)<br>Soft drinks and juices containing sugar (1 glass or 1 cup)<br>Soft drinks, diet (light) (1 glass or 1 cup)<br>Vegetables (half a cup or at the size of a tennis ball of tomato, broccoli, leafy vegetables etc)<br>Sweets (1 chocolate bar or half a cup of sweets, cookies, or ice-cream)<br>Salty snacks/fast food (e.g.,1 small hamburger, 1 small bag of chips, 1 slice of pizza)<br>Milk or milk products, unsweetened (e.g., cheese, natural yogurt)<br>Milk or milk products, sweetened (e.g., yogurt, pudding, chocolate milk)<br>Low fibre cereal and cereal products (e.g., white bread or rusk any type, refined (not whole grain) breakfast cereals, such as coco pops)<br>Whole grain cereal and cereal products (e.g., brown bread, porridge, muesli, whole grain breakfast cereal)<br><br>(child) Breakfast weekdays (Never/rarely; 1 day; 2 days; 3 days; 4 days; 5 days)<br>(child) Breakfast weekend days (Never/rarely; 1 day (Saturday or Sunday); 2 days)<br>(child) Physical Activity of 60 minutes/day weekdays (Never/rarely; 1 day; 2 days; 3 days; 4 days; 5 days)<br>(child) Physical Activity of 60 minutes/day weekend days (Never/rarely; 1 day (Saturday or Sunday); 2 days)

	(child) Screen activities weekdays (Never/rarely; 1 day; 2 days; 3 days; 4 days; 5 days)<br>(child) Screen activities weekend days (Never/rarely; 1 day (Saturday or Sunday); 2 days)
Healthy Growth	Continuous variables:<br>Dietary energy intake (Kcal/day).<br>Total moderate-to-vigorous intensity physical activity levels (min/day)<br>Organised moderate-to-vigorous intensity physical activity levels (min/day)<br>Step Count (steps/day on weekdays and weekends<br>TV watching (hours/day on weekdays and weekends)<br>Video games (hours/day on weekdays and weekends)
	Categorical variables:<br>The foods listed below are those consumed by children on a daily, weekly, or monthly basis. Respondents need to choose one of the following response choices regarding the child’s food intake: <= 1 serve per month; 2-3 serves per month; 1 serve per week; 2-3 serves per week; 4-6 serves per week; 1 serve per day; 2-3 serves per day; >=4 serves per day)<br>Fruit; Fruit Juice (Fresh); Fruit Juice (Packed); Soda; Vegetables; Cereals; Milk; Chocolate milk; Yoghurt; Fish; Chocolates; Cakes; Chips; Pasta; Fries; Red meat; Chicken; Bread; Pizza; Souvlaki; Fast food.
Energy	Continuous variables:<br>On a day that you drink fizzy drinks and fruit squash, how many glasses, cans or bottles do you drink on such a day? Glasses or small bottles (250ml); Cans (330ml); Bottles (500ml).<br>How many fizzy drinks and fruit squash did you drink yesterday? Glasses or small bottles (250ml); Cans (330ml); Bottles (500ml).<br>On a day that you drink fruit juice, how many glasses or cartons do you drink on such a day? Glasses or small cartons (250ml); Regular cartons (330ml).<br>How many fruit juices did you drink yesterday? Glasses or small cartons (250ml); Regular cartons (330ml).
	Categorical variables:<br>How many times a week do you usually drink fizzy drinks and fruit squash/ Fruit Juices? (Never; > 1 per week; 1 per week; 2-4 days per week; 5-6 days per week; Every day or more than once per day.<br>How many times a week do you usually drink fruit juice? (Never; > 1 per week; 1 per week; 2-4 days per week; 5-6 days per week; Every day or more than once per day.<br>From Monday to Friday during school weeks on how many days do you usually eat breakfast? (I never eat breakfast at school; 1 day; 2 days; 3 days; 4 days; 5 days).<br>On how many days in the weekend days (Saturday and Sunday) do you usually eat breakfast? (I never eat breakfast on the weekend; 1 weekend day; both weekend days).<br>What do you usually have for breakfast on school days? (Just a drink (milk; fruit juice; tea; hot chocolate etc); Just food (cereal; bread; sandwich; cheese etc.); Drink with cold food; Drink with hot food (sausages; bread; pied; eggs etc); Other)<br>In a TOTAL WEEK how many hours do you do this sport? (not available; 30 minutes per week; 1 hour per week; 1.5 hour per week; 2 hours per week; 2.5 hours per week; 3 hours per week; 3.5 hours per week; 4 hours per week; 4.5 hours per week; 5 hours per week)

Table S3. Potential determinants of EBRBs by study.

Study	Potential determinants of EBRBs
Genesis	Continuous variables: NA
	Categorical variables:<br>Who is responsible for child's diet? (Mother; father; grandmother; nanny; other).<br>Do you what your child consumes during the day? (Very well; well; so and so; a little, very little).<br>Do you consider your child's diet appropriate? (Very good/very healthy; Good/healthy; neither good or bad; fairly good; not good)<br>Do you consider your child's body weight as: very hight; high; normal; less than normal; much less than normal.<br>How would you characterise your child's physical activity levels? (Very high; high; normal; low; very low).<br>Characteristics of day-care centres (internal spaces (clean and tidy); external/recreational/play spaces (very good; good; neither good or bad; bad, very bad); Safety of external spaces and access to school (very good; good; neither good or bad; bad; very bad).
ToyBox	Continuous variables:<br>How much time does your child spend doing sport in a sports club per week?
	Categorical variables:<br><br>In all questions listed below the response choices are: Parent interviewed; Spouse/partner; grandparents; others.<br>Who cooks for your child?<br>Who supervises/Feeds your child?<br>Who supervises the child in doing outdoor activities?<br><br>In all questions listed below the response choices are" Strongly agree; agree; neither agree or disagree; disagree; strongly disagree.<br>My child is allowed to drink soft drinks or packaged juices whenever he/she asks for.<br>I make water always available for my child.<br>It is bad for my child to drink soft drinks/ pre-packed juice every day.<br>I encourage my child to drink water.<br>If I would like to drink soft drinks or pre-packed juices, I would try to restrain myself because of the presence of my child.<br>I am pleased with my child's water consumption.<br>My child prefers to drink soft drinks or pre-packed juices instead of water.<br>During meals, water is always available on the table.<br>I find it difficult to give my child water if he/she wants soft drinks or pre-packed juices.<br>My child does not enjoy drinking water.<br>I make soft drinks or pre-packed juices always available for my child.<br>My child's water consumption is within the appropriate recommendations.<br>I give soft drinks or pre-packed juices to my child as a reward or to comfort him/her.<br>During meals, soft drinks or pre-packed juices are always available on the table.<br>My child drinks soft drinks or pre-packed juices only on certain occasions e.g., birthdays.<br>My child likes to eat fruits or vegetables as a snack.<br>My child likes to eat dairy as a snack.<br>My child likes to eat cereals/bread as a snack.<br>I often give fruits or vegetables as snacks to my child.<br>I often give dairy as snacks to my child.<br>I often give cereals/bread as snacks to my child.<br>I make fruit or vegetables snacks regularly available for my child.

	I make dairy snacks regularly available for my child.<br>I make cereals/bread snacks regularly available for my child.<br>My child chooses sweet or salty snacks, when fruit or vegetables snacks are available.<br>My child chooses sweet or salty snacks when other children eat fruit or vegetables snacks.<br>I think eating sweet or salty snacks is not bad for my child.<br>I make sweet or salty snacks regularly available for my child.<br>My child is not allowed to snack while watching TV.<br>My child is allowed to eat fruits or vegetables as snacks without asking.<br>My child is allowed to eat dairy or cereals/bread as snacks without asking.<br>My child is allowed to eat sweet or salty snacks only at certain occasions i.e., birthdays.<br>I give sweet or salty snacks to my child as a reward or to comfort him/her.<br>If I prohibit my child to eat a sweet or salty snack, I find it difficult to stick to my rules if he/she starts nagging.<br>I find it difficult to restrain myself from eating sweet or salty snacks because of the presence of my child.<br>I am pleased with my child's snacking behaviour.<br>My child likes to be physically active.<br>My child enjoys taking part in sports.<br>Being physically active is good for my child.<br>I plan physical activity for my child on a regular basis.<br>I find it difficult to organise our family so that we have enough time for active transport.<br>Toys or equipment/material (ball, rope, bike, swing, …) are available for my child to play actively outside or inside.<br>I find it difficult to let my child be physically active if I want my child to be quiet so that I can do my household or work.<br>I find it difficult to let my child be physically active if the weather conditions are bad or it is very cold/hot outside.<br>I find it difficult to let my child be physically active outside as I always have to be there to supervise him/her.<br>I encourage my child to be physically active.<br>I like doing physical activities together with my child.<br>I reward my child or comfort him/her by being physically active together with him/her.<br>I find it difficult to insist that my child is physically active if he/she does not want to and starts nagging.<br>I try to be physically active together with my child regularly.<br>My child is allowed to run around and be physically active inside our house.<br>I am pleased with my child's physical activity level.<br>I think screen viewing activities are beneficial and educational for my child.<br>My child likes to watch TV/DVD/Video.<br>My child prefers to watch TV for a long time instead of doing other activities.<br>I find it difficult to limit my child's screen viewing activities if he/she does not want to and starts nagging.<br>I like watching TV/DVD/Video together with my child.<br>I make sure that there are other activities available for my child to do instead of screen viewing.<br>My child does not like to do activities while standing up.<br>My child's TV viewing levels are within the appropriate recommendations.<br>I think it is necessary to limit the screen viewing activities for my child.<br>I encourage my child to do something else instead of watching TV/DVD/Video.<br>It is a habit to organise my family so that we can see programs we like at TV.

	I try to restrain myself from watching TV/DVD/Video while my child is present.<br>My child is allowed to watch TV for as long as he/she wants.<br>I punish my child by forbidding him/her to watch TV.<br>I do not think it is necessary to limit TV viewing for my child if he/she looks at the appropriate children's programs.<br>I am pleased with my child's screen viewing activities.<br><br>What do you think about your child's weight? (Very low; low; not too low/not too high; high; very high)<br>Is your child member in a sports club? (Yes, No)<br>What kind of sport does your child do in a sports club? (Football; preschool gymnastics; swimming; dance; tennis; martial arts; ballet; Other).
Feel4Diabetes	Continuous variables: NA
	Categorical variables:<br>Parents' responses to all questions below are: Always; Often; Sometimes; Rarely; Never.<br>Fruits availability<br>Fruit juices freshly squeezed or prepacked without sugar availability.<br>Fruit juices, prepacked, containing sugar availability.<br>Soft drinks containing sugar availability.<br>Soft drinks without sugar availability<br>Vegetables availability<br>Sweets, biscuits, ice cream, cakes, pastries availability<br>Salty snacks (e.g., chips, savoury pastries) availability<br><br>Parents' responses to all questions below are: Very often; Often; Sometimes; Rarely; Never.<br>How often do you consume fresh fruits with your child?<br>How often are you physically active with your child?<br>How often do you watch TV together with your child?<br>How often do you allow your child to eat sweets and/or salty snacks whenever he/she asks for?<br>How often do you allow your child to watch TV or DVD when he/she wants?<br>How often do you allow your child to use the computer, mobile or tablet when he or she wants?<br>How often do you reward your child by allowing him/her to watch TV/DVD or use the computer, mobile or tablet?<br>How often do you reward your child with sweets, salty snacks (e.g., potato chips) or fast food?<br>How often do you reward your child by being physically active together with him/her or by taking him/her to the playground or to the park?<br><br>Parents' responses to all questions below are: Yes; No<br>TV availability in child's room<br>DVD player availability in child's room<br>Game console (e.g., PlayStation) availability in child's room<br>Computer availability in child's room<br>Tablet or smartphone availability in child's room
Healthy Growth	Continuous variables:<br>Places near the house where the child can play (times per week)<br>▪ Parks; Playgrounds; Pavement/Pedestrian zone; Street; Open fields; School yards; Public sport clubs/centres; Private gyms.
	Categorical variables:<br>All variables listed below refer to parental levels of satisfaction on their children's diet. Parents' responses to all relevant questions are: Much less than it should; Less than it should; Just as much as it should; More than it should; Much more than it should.

	▪ I believe that the quantity of fruits my child consumes are:<br>▪ I believe that the quantity of vegetables my child consumes are:<br>▪ I believe that the quantity of dairies my child consumes are:<br>▪ I believe that the quantity of meat my child consumes are:<br>▪ I believe that the quantity of legumes my child consumes are:<br>▪ I believe that the quantity of soda my child consumes are:<br>▪ I believe that the quantity of chocolate or other sweets my child consumes are:<br>▪ I believe that the quantity of chips my child consumes are:<br><br>All variables listed below refer to the availability of foods at home. Parents' responses to all relevant questions are: <= 1 time per month; 2-3 times per month; 1-2 times per month; 3-4 times per month; 5-6 times per month; Every day).<br>▪ Fruit; Fruit Juice (Fresh); Fruit Juice (Packed); Soda; Vegetables; Cereals; Milk; Chocolate milk; Yoghurt; Fish; Chocolates; Cakes; Chips; Pasta; Fries.<br><br>All variables listed below refer to neighbourhood safety. Parents' responses to all relevant questions are: Strongly agree; agree; neither agree or disagree; disagree; strongly disagree.<br>There are sidewalks without obstacles in my neighbourhood, parks and infrastructures that make exercise and walking easy.<br>There is too much traffic in the streets of my neighbourhood that make walking and exercise very difficult.<br>There is a high crime rate in my neighbourhood.<br>It is safe for my child to play, walk or bike in our neighbourhood.<br>It is much safer if my child being at home than playing outside in the neighbourhood.<br>I like my neighbourhood. It has nice aesthetics and street layout.<br><br>All variables listed below refer to food insecurity questions. Parents' responses to all relevant questions are: Yes; No; I don't know.<br>Not enough money for food?<br>Not enough food availability for shopping?<br>Not enough time for shopping or cooking?<br>Too hard to get to the store?<br>On a diet?<br>Not working stove available?<br>Not able to cook or eat because of health problems?<br><br>Parental estimation of their child's weight status (Underestimation; Correct estimation; Overestimation).<br>Children pester/nag power over their father/mother (Always; Usually; Often; Sometimes; Rarely; Never)<br>Do parents limit their children in the time they watch or play with video games? (No; Yes)<br>Do parents forbid/limit the consumption of sweets or beverages from their children? (No; Yes)
Energy	Continuous variables: NA
	Categorical variables:<br>Children's' responses to all questions below are: Very bad; bad; neither good or bad; good; very good<br>I think that drinking fizzy drinks or fruit squash is…<br>If I drink fizzy drinks or fruit squash, my parents/care givers think this is…<br>If I drink fizzy drinks or fruit squash, most of my friends think this is…<br><br>Children's' responses to all questions below are: I fully disagree; I disagree a bit; neither agree or disagree; I agree a bit; I fully agree.

	I think drinking fizzy drinks or fruit squash will make me fat.<br>I like the taste of fizzy drinks or fruit squash. recoded<br>▪ Drinking fizzy drinks or fruit squash is something that I do without even really thinking about it. recoded<br><br>Children's' responses to all questions below are: Never; Not often; Sometimes; Often; Always.<br>How often do your parents/care givers drink fizzy drinks or fruit squash?<br>How often do most of your friends drink fizzy drinks or fruit squash?<br>If I ask my parents/care givers for a fizzy drink or fruit squash, I get one. recoded<br>I am allowed to take fizzy drinks or fruit squash, whenever I want.<br>If you ask your parents/care givers to buy a certain brand of fizzy drink or fruit squash, will they do it?<br>Are there usually fizzy drinks or fruit squash at your home?<br><br>I find drinking no fizzy drinks or fruit squash… (Very difficult; difficult; Neither easy or difficult; Easy; Very easy).<br>Do your parents/care givers have rules about how many fizzy drinks or fruit squash you are allowed to drink? (No; Yes)<br><br>Children's responses to the following question "In which situations do you usually drink fizzy drinks or fruit squash? During the weekend.; Breakfast; Lunch; Dinner; At school; While watching TV; Between meals; During/after sports; When I am with friends; At birthdays/parties". Children's' responses to the different situations listed below are" are: No; Yes.<br>I think that drinking fruit juice is… (Very bad; bad; neither good or bad; good; very good)<br>Do your parents/care givers have rules about how many fruit juices you are allowed to drink? (No; Yes)<br>Are there usually fruit juices at your home? Never; Not often; Sometimes; Often; Always.<br><br>Children's responses to the following question "In which situations do you usually drink fruit juice? During the weekend.; Breakfast; Lunch; Dinner; At school; While watching TV; Between meals; During/after sports; When I am with friends; At birthdays/parties". Children's' responses to the different situations listed below are" are: No; Yes.<br><br>Children's' responses to all questions below are: Very bad; bad; neither good or bad; good; very good.<br>I think eating breakfast is…<br>If I eat breakfast, my parents/care givers/ my friends think this is...<br><br>Children's' responses to all questions below are: I fully disagree; I disagree a bit; neither agree or disagree; I agree a bit; I fully agree.<br>I think NOT eating breakfast will make me fat.<br>My parents/care givers encourage me to eat breakfast…<br>Eating breakfast is something that I do without even really thinking about.<br><br>Children's' responses to all questions below are: Never; Not often; Sometimes; Often; Always.<br>How often do your parents/care givers/most of your friends eat breakfast?<br>If you ask your parents/care givers to buy a certain brand of food or drink for breakfast, will they do it?<br>Are there usually breakfast products (milk, cereals, bread etc) at your home?<br>How often do you eat breakfast with your parents/ care givers?<br><br>What is the reason you usually skip breakfast? (I never skip breakfast; I do not have a enough time; I do not like breakfast at home; I have never thought about it; I am not hungry in the morning.; I just cannot eat early in the morning).

	I think it is recommended for children my age ... (To skip breakfast; Eat breakfast if you feel like it; Eat breakfast on schooldays; Eat breakfast every day; I don't know what is recommended).<br>I find eating breakfast everyday… (Very difficult; difficult; Neither easy or difficult; Easy; Very easy).<br>Do your parents/care givers have rules about whether you should eat breakfast? (No; Yes)<br><br>Children's responses to the following question "In which situations do you usually eat your breakfast? At home.; In bed; While watching TV; On my way to school; At school before the class starts; I never eat breakfast". Children's' responses to the different situations listed below are" are: No; Yes.<br><br>Children's' responses to all questions below are: Very bad; bad; neither good or bad; good; very good.<br>I think that physical activity/sports is…<br>If I do physical activity/sports, my parents/care givers think this is…<br>If I do physical activity/sports, most of my friends think this is…<br><br>Children's' responses to all questions below are: I fully disagree; I disagree a bit; neither agree or disagree; I agree a bit; I fully agree.<br>I think NOT doing physical activity/sports will make me fat…<br>I like doing physical activity/sports.<br>Doing physical activity/sports is something that I do without even really thinking about…<br>My parents/care givers encourage me to do physical activity/sports…<br>My parents/care givers help me if I need something for my sports…<br><br>Children's' responses to all questions below are: Never; Not often; Sometimes; Often; Always.<br>How often do most of your friends do physical activity/sports?<br>If you indicate that you like a certain physical activity/sport will your parents/care givers allow you to do it?<br>How often do you take part in physical activity/ do sports with your parents care givers?<br><br>Children's' responses to all questions below are: No; Yes.<br>Do your parents/care givers have rules about whether you should be physically active/do sports?<br>Do your parents/care givers allow you to take part in physical activity/do sports?<br>Do you have the following things at home that you can use for physical activities/sports? Bike; Tennis and/or badminton racket; Ball (basketball, volleyball, football etc); Sporting shoes; Skipping rope; Skates; Skis; Skateboard.<br><br>I think it is recommended for children my age to be active… (Once per week; some days per week; every day for 30 min; every day for 1 hour; every day for 2 hours; every day for 3 to 4 hours; I do not know what is recommended)<br><br>Children's' responses to all questions below are: Not at all; 30 min per day; 1 hour per day; 1.5 hours per day; 2 hours per day; 2.5 hours per day; 3 hours per day; 3.5 hours per day; 4 hours per day.<br>About how many hours a day do you usually watch television in your free time? All weekdays<br>About how many hours a day do you usually watch television in your free time? All weekend days<br>About how many hours a day do you usually play games on a computer, or use your computer for leisure activity in your free time? All weekdays<br>About how many hours a day do you usually play games on a computer, or use your computer for leisure activity in your free time? All weekend days<br>About how many hours did you watch television yesterday?

	About how many hours a day did you play games on a computer, or use your computer for leisure activity yesterday?<br><br>Children's' responses to all questions below are: Very bad; bad; neither good or bad; good; very good.<br>I think that tv viewing is…<br>If I watch television, my parents/care givers think this is…<br>If I watch television, most of my friends think this is…<br><br>Children's' responses to all questions below are: Never; Not often; Sometimes; Often; Always.<br>How often do your parents/care givers watch television?<br>How often do most of your friends watch television?<br>If I ask my parents/care givers to watch television, I can do so.<br><br>Children's' responses to all questions below are: I fully disagree; I disagree a bit; neither agree or disagree; I agree a bit; I fully agree.<br>I think watching too much television will make me fat.<br>I like watching television.<br>Watching television is something that I do without even really thinking about.<br>My parents/caregivers allow me to watch television, whenever I want.<br><br>Children's' responses to all questions below are: Never; < once per week; Once a week; 2-4 days per week; 5-6 days per week; every day; more than once per day.<br>How often do you watch television with your parents care givers?<br>How often do you watch television during meals? Breakfast<br>How often do you watch television during meals? Lunch<br>How often do you watch television during meals? Dinner<br><br>Children's' responses to all questions below are: No; Yes<br>Do your parents/care givers have rules about how many hours per day you are allowed to watch television?<br>Do you have a television in your bedroom?<br><br>Parents' responses to all questions below are: Never; Not often; Sometimes; Often; Always.<br>There are soft drinks available at home for my child.<br>I pay attention to the number of soft drinks that my child drinks.<br>If my child asks for soft drinks, I will give it to him/her.<br>My child is allowed to take soft drinks, whenever (s)he wants.<br>I negotiate with my child how much soft drinks (s)he is allowed to drink.<br>How often do you tell your child that soft drinks are not good for him/her.<br>How often do you tell your child that soft drinks can make him/her fat?<br>How often do you tell your child that soft drinks are bad for his/her teeth?<br>If I would like to dink soft drinks, I would restrain myself because of the presence of my child.<br>If I prohibit my child from drinking soft drinks, (s)he tries to drink it anyway.<br>If I prohibit my child from drinking soft drinks, I find it difficult to stick to my rule(s), if (s)he starts negotiating.<br>I give soft drinks to my child as a reward or to comfort him/her.<br>How often do you or your spouse drink soft drinks together with your child?<br>There are fruit juices available at home for my child.<br>I pay attention to the amount of fruit juices that my child drinks.<br>If my child asks for fruit juices, I will give it to him/her.<br>My child is allowed to take fruit juices, whenever (s)he wants.<br>I negotiate with my child how much fruit juices (s)he is allowed to drink.

	How often do you tell your child that fruit juices are not good for him/her.<br>How often do you tell your child that fruit juices can make him/her fat?<br>How often do you tell your child that soft drinks are bad for his/her teeth?<br>If I would like to drink fruit juices, I would restrain myself because of the presence of my child.<br>If I prohibit my child from drinking fruit juices, (s)he tries to drink it anyway.<br>If I prohibit my child from drinking fruit juices, I find it difficult to stick to my rule(s), if (s)he starts negotiating.<br>I give fruit juices to my child as a reward or to comfort him/her.<br>How often do you or your spouse drink fruit juices together with your child?<br>There are breakfast products (milk, cereals, bread etc) available at home for my child.<br>I encourage my child to have breakfast.<br>I pay attention what kind of products my child is eating for breakfast.<br>My child is allowed to skip breakfast.<br>I negotiate with my child on how much …<br>How often do you tell your child that eating breakfast is good for you.<br>If I prohibit my child from skipping breakfast, (s)he tries to skip it anyway.<br>If I prohibit my child from skipping breakfast, I find it difficult to stick to my rule(s) if (s)he starts negotiating.<br>I praise my child if (s)he eats breakfast.<br>How often do you eat breakfast with your child?<br>I pay for my child to take part in physical activity /sports.<br>I bring my child to physical activity/sports sessions.<br>I encourage my child to take part in physical activity /sports.<br>I pay attention that my child does enough physical activity/sports.<br>My child is allowed to skip physical activity /sport sessions whenever (s)he wants.<br>My child is allowed to skip physical activity /sport sessions whenever (s)he wants.<br>I negotiate with my child how much physical activity…<br>How often do you tell your child that physical activity/ sports are good for him/her.<br>If I try to prohibit my child from not taking part in a physical activity/sport session, (s)he will try to skip it anyway.<br>If I try to prohibit my child from skipping a physical activity/sport session, I find it difficult to stick to my rule(s) if (s)he starts negotiating.<br>I praise my child if (s)he takes part in physical activity /sports.<br>I punish my child by not allowing him/her to take part in his/her physical activity sessions/sports.<br>In general, how often do you watch television during the following meals? – Breakfast.<br>TV/video/DVD is available in my Childs's room.<br>I pay attention to the amount of time my child watches TV/video/DVD.<br>If my child asks if (s)he is allowed to watch TV/video/DVD, I will allow it.<br>My child is allowed t watch TV/video/DVD whenever (s)he wants.<br>I negotiate with my child how much TV/video/DVD (s)he is allowed to watch.<br>How often do you tell your child that watching TV/video/DVD is not good for him/her?<br>How often do you tell your child that watching TV/video/DVD can make him/her fat?<br>How often do you tell your child that watching TV/video/DVD is bad for him/her sight?<br>If I would like to watch TV/video/DVD i would restrain myself because of her presence of my child?<br>If I prohibit my child watching TV/video/DVD, (s)he tries to watch anyway.<br>If I prohibit my child from watching TV/video/DVD I find it difficult to stick to my rule(s) if (s)he starts negotiating.<br>I allow my child to watch TV/Video/DVD as a reward or to comfort him/her. recoded<br>How often do you (one parent/spouse/partner or both) watch television together with your child?

Table S4. Socio-economic status variables used for the identification of vulnerable populations of "children in need".

Study	Stratification factors
Genesis	Continuous variables:<br>Household total annual income<br>Father/Mother age (years)<br>Father/Mother body weight, height & BMI
	Categorical variables:<br>Child's Sex<br>Urbanisation degree (metropolitan; urban; rural areas)<br>Father/Mother country of birth.<br>Father/Mother born abroad.<br>Father/Mother ethnicity<br>Father/Mother level of education<br>Father/Mother occupational status<br>Father/Mother weight status(Underweight; Normal weight; Overweight; Obese)
ToyBox	Continuous variables:<br>Socio-economic status (SES) code (Low; Medium; High)<br>Father/Mother age (years)<br>Father/Mother body weight, height & BMI
	Categorical variables:<br>Child's Sex<br>Father/Mother country of birth.<br>Ethnicity (based on language spoken at home)<br>Father/Mother/Carer level of education<br>Father/Mother/Carer occupational status<br>Father/Mother weight status (Underweight; Normal weight; Overweight; Obese)
Feel4Diabetes	Continuous variables:<br>Father/Mother age (years)<br>Father/Mother body weight, height & BMI
	Categorical variables:<br>Father/Mother level of education (< 6 years; 7-9 years; 10-12 years; 13-14 years; 15-16 years; > 16 years)<br>Father/Mother main occupation (Stay at home parent; Work full-time; Work part-time; Unemployed; Full time student; Retired; Other)<br>Father/Mother weight status (Underweight; Normal weight; Overweight; Obese)
Healthy Growth	Continuous variables:<br>Father/Mother age (years)<br>Father/Mother education (years)<br>Family size (no. of family members)
	Categorical variables:<br>Child's Sex<br>Urbanization degree (urban, semi-urban, rural region of residence).<br>Socio-economic level of participating schools (Lower; Medium; Higher)<br>Annual family income (<12K Euros; 12L-20K Euros; 20-30K Euros; 30-40K Euros; >40K Euros)<br>House ownership (Yes/No)<br>Father/Mother ethnicity<br>Father/Mother employment status (Employed full time; Employed part time; Unemployed; Retired)<br>Father/Mother weight status (Underweight; Normal weight; Overweight; Obese)
Energy	Continuous variables:

	Father/Mother age (years)<br>Father/Mother body weight, height & BMI
	Categorical variables:<br>Child’s Sex<br>Urbanization degree (1st Tertile; 2nd Tertile; 3rd Tertile).<br>Father/Mother ethnicity (Based on Language spoken at home).<br>Parents’ marital status (Single; married; living with my parent, but not married; separated; divorced; other)<br>Father/Mother main occupation (Employee in public sector; employee in private sector; self-employed; no paid job)<br>Father/Mother level of education (< 7 years; 7-9 years; 10-11 years; 12-13 years; >= 14 years)<br>Father/Mother weight status (Underweight; Normal weight; Overweight; Obese)
